# Lawson Tait’s Wonderful Success in Ovariatory

**Published:** 1886-07

**Authors:** 


### LAWSON TAIT'S WONDERFUL SUCCESS IN OVARIOTOMY.

A phenomenon of the nineteenth century is the progress made in abdominal surgery as evidenced by Lawson Tait's one hundred and thirty-nine consecutive successful laparotomies for the removal of the ovaries or tubes or both.

This progress, like many others, has been achieved, however, only by a revolution ; and it has brought about a revolution in pathology and practice, the discarding of old ideas—orthodox for centuries, and the adoption of the very reverse The world sewed with the eye in the head of the needle for centuries; Howe put it in the point of the needle and the sewing machine was born. Tait discarded opium in peritonitis and now gives purgatives, a thing which formerly would have condemned any man of malpractice in any court of the United States. The fear of the peritoneum was what retarded progress. "It's the peritonitis that beats us," said Baker Brown. Tait abandoned fear and now "beats peritonitis." He boldly invades the "sacred sac," having arrived at the conclusion that "we are justified in opening the peritoneum very much as we open our pockets;" and that "suppurative peritonitis may be treated like any other abscess."—*Lawson Tait in Brit. Med. Journal.*

His experience has done away with several other fallacies, and the result, as formulated by himself into a rule of action is

—"When in doubt, open the abdomen." The rule formerly was, we believe, with the average general practitioner—"when in doubt, give calomel"—the parallel of "leading trumps," according to Hoyle. Tait has "coppered" on opium; it is no longer "trumps."

Perhaps the most striking feature in the report of his one hundred and thirty-nine cases, is the fact that Mr. Tait uses no kind of antiseptic or germicide. This would look like a deadener to the idea of septic germs altogether. He shows his contempt for germs in the following language: "The water I use is plain unfiltered tap-water, warmed by the addition of enough from the boiler. It is full of germs and spores and small beasts of thirty-four different varieties, according to a careful report of Dr. Alford Hill," [*loc. cit.*] and again, "My fear of germs has steadily diminished, so that if I could get them in sufficiently large quantities I would willingly stuff my pads with them instead of wool."

It is to be remarked that of his one hundred and thirty-nine cases not one had been tapped; and Mr. Tait says: "and for many years past I have not had a fatal case that had not been tapped." He concludes, therefore, very reasonably that "in Stilling's strong words tapping an ovarian tumor is a surgical crime."

Another element in the wonderful success of this great and bold operator is the reduction of the length of the incision. He says: "Nothing save an irreducible solidity can justify a five inch incision;" and that he regards any incision over three inches as excessive. But "the main feature of change accomplished within the last eight years in ovariotomy," says Mr. Tait, "is the treatment of the pedicle. The clamp has been wholly deserted and the intraperitoneal method adopted." He prefers the silk suture to the cautery, on account of its universal applicability, and uses the "Staffordshire knot" which, he says, has never failed him. Mr. Tait also insists on including in the suture all tissues divided–keeping the corresponding tissues on each side correctly in relation to each other ; saying : "If you exclude tendon and muscle you exclude the chief elements of strengh in the union."

Thus is born another era in Surgery--and Tait is immortal!

